# GluN2A and GluN2B subunit-containing NMDA receptors in hippocampal plasticity

**DOI:** 10.1098/rstb.2013.0163

**Published:** 2014-01-05

**Authors:** Olivia A. Shipton, Ole Paulsen

**Affiliations:** 1 Department of Physiology, Development and Neuroscience, University of Cambridge, Downing Street, Cambridge CB2 3EG, UK; 2 Department of Physiology, Anatomy and Genetics, University of Oxford, Oxford OX1 3QX, UK

**Keywords:** NMDA receptor subunit, hippocampus, plasticity, learning

## Abstract

*N*-Methyl-d-aspartate receptor (NMDAR)-dependent synaptic plasticity is a strong candidate to mediate learning and memory processes that require the hippocampus. This plasticity is bidirectional, and how the same receptor can mediate opposite changes in synaptic weights remains a conundrum. It has been suggested that the NMDAR subunit composition could be involved. Specifically, one subunit composition of NMDARs would be responsible for the induction of long-term potentiation (LTP), whereas NMDARs with a different subunit composition would be engaged in the induction of long-term depression (LTD). Unfortunately, the results from studies that have investigated this hypothesis are contradictory, particularly in relation to LTD. Nevertheless, current evidence does suggest that the GluN2B subunit might be particularly important for plasticity and may make a synapse bidirectionally malleable. In particular, we conclude that the presence of GluN2B subunit-containing NMDARs at the postsynaptic density might be a necessary, though not a sufficient, condition for the strengthening of individual synapses. This is owing to the interaction of GluN2B with calcium/calmodulin-dependent protein kinase II (CaMKII) and is distinct from its contribution as an ion channel.

## 1. Introduction

Deciphering how neuronal networks can robustly store information after even a single learning episode has proved one of the biggest challenges in neuroscience. The best-supported cellular model for learning and memory proposes that pertinent neuronal activity leads to long-lasting changes in synaptic weights distributed throughout the network [1]. Such ‘synaptic plasticity’ has been extensively studied at hippocampal excitatory synapses, where most forms of plasticity require the activation of a particular type of glutamate receptor known as the *N*-methyl-d-aspartate receptor (NMDAR) for their induction [2]. NMDARs are particularly attractive as molecular mediators of plasticity because of their Ca^2+^ permeability and also their coincidence detector properties that result from a voltage-dependent Mg^2+^ block [3]. Plasticity encompasses both increases (long-term potentiation, LTP) and decreases (long-term depression, LTD) in synaptic strength and is expressed by changes in α-amino-3-hydroxy-5-methyl-4-isoxazolepropionic acid receptors (AMPARs) at the postsynaptic element and/or changes in presynaptic transmitter release. Ultimately, such changes appear to be consolidated by structural alterations. Nevertheless, how relevant neuronal activity triggers meaningful plasticity *in vivo* remains uncertain. Moreover, if plasticity does support learning and memory, it is not yet clear whether a learning episode triggers both potentiation and depression in parallel across different sets of synapses or, alternatively, primarily one or the other.

This review addresses the controversial field around the hypothesis that NMDARs with distinct subunit compositions are responsible for the induction of the two opposing forms of plasticity, and that altering this subunit balance is an important mechanism to fine-tune synaptic strength. Firstly, it outlines how different subunit compositions affect the functional and dynamic properties of the NMDAR and the implications for its role in signal transmission. Secondly, it assesses whether these NMDAR subunits contribute to distinct forms of plasticity. Finally, as NMDAR-mediated plasticity is thought to be involved in learning and memory [4,5], it summarizes the evidence that different types of learning and memory might be supported by NMDARs with distinct subunit compositions. Several mechanisms might contribute to plasticity in different brain regions, and some of these are NMDAR independent, such as LTP at the mossy fibre synapse in CA3 [6] and metabotropic glutamate receptor-dependent LTD in CA1 [7]. Furthermore, plastic changes at the synapse can have both short- and long-term components [8], and these may be supported by distinct processes and have different NMDAR subunit-dependency for their induction [9]. This review focuses on the NMDAR-dependent forms of long-term plasticity in the hippocampus, in particular at the CA3-CA1 synapse, to facilitate comparison between experiments and because this synapse in particular has been implicated in hippocampus-dependent associative learning.

## 2. The hypothesis

A number of theories have been proposed to explain how different patterns of neuronal activity can lead to opposite effects on synaptic strength when both forms of plasticity depend on the same type of receptor. A unifying aspect of the postsynaptic-expression theories is that NMDAR-mediated Ca^2+^ influx at the postsynaptic element, the ‘spine’, must ultimately be coupled to the different intracellular signalling cascades that mediate changes in synaptic weights.

It was quickly identified that the magnitude of Ca^2+^ influx through NMDARs in the traditional high-frequency paradigms used to induce LTP was much greater than that during the low-frequency LTD paradigms. Therefore, it was proposed that different levels of Ca^2+^ influx could couple to distinct intracellular signalling pathways to cause the molecular, and ultimately structural, changes at the synapse that underlie the two directions of plasticity [10]. This was also extended to the induction of plasticity by precise spike timing [11]. However, it seemed that a mechanism based on Ca^2+^ levels alone may not be sufficiently robust, and it also could not account for some experimental observations; thus, Ca^2+^ time-course was suggested to be important [12,13]. Postsynaptic Ca^2+^ dynamics are tightly regulated by small-conductance Ca^2+^-activated K^+^ channels [14,15], voltage-dependent Ca^2+^ channels [16,17] and intracellular Ca^2+^ release [18]; the Ca^2+^ microdomains that these generate could provide a finer level of control in the regulation of bidirectional plasticity.

In parallel to refinements in the theory linking Ca^2+^ dynamics and bidirectional plasticity arose the suggestion that the NMDAR itself could intrinsically dissociate synaptic strengthening and weakening in response to activity patterns. The distinct kinetic properties conferred by NMDAR subunits might allow the NMDAR composition to exert a finer level of control over the postsynaptic Ca^2+^ dynamics. They also make distinct molecular associations, and thus could independently couple to the downstream kinase and phosphatase pathways that have been established to regulate each direction of plasticity (for review, see [19]). Furthermore, the relative abundance of these subunits at synaptic sites is tightly regulated throughout development, and a transition in subunit dominance correlates with changes in the ease of plasticity induction [20]. All this evidence converged on the attractive suggestion that the type of plasticity induced could be determined by the type of NMDAR subunit activated, perhaps by enabling transmission of distinct signals and tightly coupling them to different downstream signalling molecules. However, the findings from studies that set out to investigate this hypothesis have been inconclusive.

## 3. NMDA receptors

**Figure 1.**
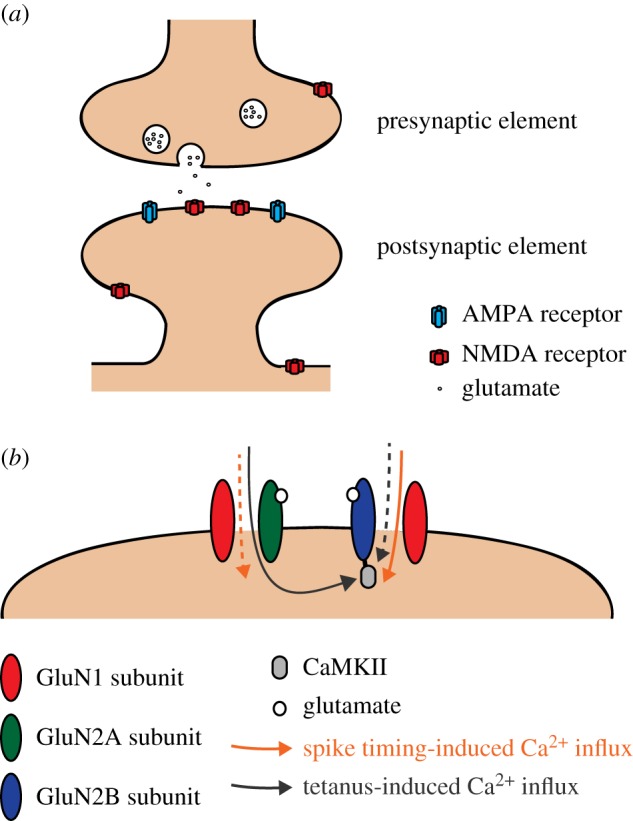
NMDA receptor location and subunits in synaptic plasticity. (*a*) NMDARs are found both pre- and postsynaptically, and these two NMDAR populations might play different roles in synaptic plasticity. In the postsynaptic membrane, NMDARs are found synaptically, perisynaptically and extrasynaptically, where they are also likely to perform different functions. (*b*) During induction of spike timing-dependent LTP, Ca^2+^ influx through GluN2B subunit-containing NMDARs (orange arrow) directly activates CaMKII to trigger LTP. Tetanic activation elicits a larger Ca^2+^ influx through GluN2A subunit-containing NMDARs (grey arrows), which reaches and activates CaMKII anchored at the postsynaptic density (PSD) by the C-terminal of the GluN2B subunit. In both cases, it is CaMKII activation that triggers downstream signalling cascades mediating LTP expression, suggesting that the presence of the GluN2B subunit at the PSD is important for LTP induction irrespective of whether it supports a majority of the Ca^2+^ influx.

NMDARs are found both pre- and postsynaptically (figure 1*a*), and evidence from neocortical areas has set a precedent that these distinct populations of NMDARs may support different plasticity mechanisms [21–23]. This might also apply in the hippocampus, because plasticity can be mediated by changes in presynaptic release probability under some experimental conditions [24–26]; for further discussion of presynaptically expressed hippocampal LTP, readers are directed to a recent review by Bliss & Collingridge [27]. Our review focuses on postsynaptic NMDARs, which can be found synaptically, perisynaptically and extrasynaptically. These three populations of receptors are recruited differentially by neuronal activity patterns, suggesting that they may play distinct functional roles; however, it remains controversial as to how subunit composition and synaptic location relate.

NMDARs consist of two obligatory GluN1 subunits and two additional GluN2 or GluN3 subunits that confer the particular properties of the receptor. Each subunit consists of four major domains: the N-terminal domain containing binding sites for allosteric modulators, such as Zn^2+^ and ifenprodil; the agonist-binding domain, where the binding sites for glycine/d-serine (on GluN1) and glutamate (on GluN2) are located and where competitive antagonists act; the pore domain, accessible to pore blockers, such as phenycyclidine (PCP) and MK801, and, lastly, the C-terminal domain (CTD), which binds to different intracellular mediators. The two predominant GluN2 subunits in the hippocampus are GluN2A and GluN2B, although GluN2C and GluN2D are also present, in particular early in development, but also in low quantities in adulthood [28]. Hippocampal NMDARs can be diheteromeric (GluN1/GluN2A and GluN1/GluN2B) or triheteromeric (GluN1/GluN2A/GluN2B). The expression of GluN2B is high at birth but decreases into adulthood, while GluN2A expression increases with age [20,28–30]. The triheteromeric population is increasingly recognized to form a large proportion of the synaptic NMDARs in the adult brain [9,31–33], but, as these receptors cannot be selectively interrogated pharmacologically, the characteristics of triheteromeric receptors can only be inferred. Thus, their precise role remains enigmatic, though likely important.

### (a) Functional and dynamic properties

Measurements of channel currents or postsynaptic responses to single stimuli have revealed notable differences in the kinetics of GluN2A and GluN2B subunit-containing NMDARs, and this is one way in which these channel subtypes could influence the induction of plasticity. Single-channel recordings in human embryonic kidney (HEK) cells showed that GluN1/GluN2A receptors have a higher probability of opening in response to glutamate and also a higher peak open probability than GluN1/GluN2B receptors [34]. These findings have been supported by whole-cell measurements in HEK cells [35] and also acute hippocampal slices [31] where channel open probability was estimated by use-dependent block of NMDARs with MK801. The faster activation and deactivation rates of individual NMDARs comprising GluN1/GluN2A result in whole-cell currents that rise and decay more quickly than those supported by GluN1/GluN2B, and a triheteromeric population apparently has an intermediate decay time constant [36].

The response of NMDARs to multiple stimuli at a range of frequencies over different durations is particularly important in the context of synaptic plasticity. Measurements from single-channel recordings have been used to simulate the responses of GluN2A and GluN2B subunit-containing NMDARs to trains of presynaptic activity and this showed a relationship between stimulation frequency and total charge transfer that differed between the two subunits [34]. It was found that at the very low frequencies (0.1–0.3 Hz) used in some plasticity protocols, GluN1/GluN2B channels supported twice as much charge transfer owing to their slower deactivation rate. At the 1 Hz stimulation favoured for LTD induction, charge transfer through GluN1/GluN2B channels still exceeded that through GluN1/GluN2A, but to a lesser extent, and it was equivalent between channels at 2 Hz. Modelling also revealed that the duration of activity is important at high frequencies of stimulation; for the 100 Hz frequency often used in high-frequency induction protocols, the duration of the stimulus train dramatically changed the charge transfer through GluN2A and GluN2B subunit-containing NMDARs. The two channel subtypes permitted equivalent charge transfer for 100 ms of stimulation; however, for a 1000 ms stimulus train, the typical duration used to induce LTP, GluN1/GluN2A supported twice the charge transfer of GluN1/GluN2B [34]. Desensitization of GluN2A and GluN2B subunit-containing NMDARs will also contribute to their ability to mediate charge transfer, but the effect will be highly influenced by the subunit composition at the postsynaptic spine; modelling has suggested that a larger fraction of GluN2A subunit-containing NMDARs will be desensitized at stimulation frequencies from 5 to 100 Hz, but that the difference between GluN2A and GluN2B will be the lowest at 100 Hz for 1000 ms [37]. Although these modelling studies provide useful information predicting how subunit composition and plasticity protocols may interact, further experimental investigation is essential to test their predictions under physiological conditions.

It is generally assumed that the magnitude of Ca^2+^ influx is related to charge transfer, which suggests that differences in charge transfer between channel subtypes would be important in their ability to trigger distinct forms of plasticity that require different postsynaptic Ca^2+^ dynamics. However, the relationship may be more complex, because Ca^2+^ imaging of spines has revealed that the magnitude of Ca^2+^ influx in response to glutamate application does not necessarily correlate with NMDAR-mediated charge transfer [38]. Moreover, a GluN2B subunit-selective antagonist caused a greater reduction in Ca^2+^ influx in those spines that supported a higher change in Ca^2+^ per uncaging-evoked excitatory postsynaptic current (EPSC) which led the authors to conclude that GluN2B subunit-containing NMDARs support a greater Ca^2+^ influx per unit of current [38]. This had previously not been observed in recombinant GluN2A or GluN2B subunit-containing NMDARs in heterologous systems [28], which may be owing to methodological differences, the presence of triheteromeric NMDARs and/or posttranslational modifications of NMDARs in acute brain slices, and so further investigation is required. Nevertheless, if this higher Ca^2+^ influx through GluN2B subunit-containing NMDARs holds, then it suggests that this subunit could exert a disproportionate influence on plasticity.

### (b) Intracellular associations of NMDA receptors

Other ways in which NMDAR subunits could influence the direction of plasticity relate to their long CTDs, as these allow for many intracellular interactions and modifications by phosphorylation. Many of these direct and indirect molecular associations are unique, as the CTD shows a high level of sequence divergence between GluN2A and GluN2B. This permits separate regulation of their presence or absence at the synapse and could also couple each subunit to different downstream signalling cascades that support either LTP or LTD; a few key examples of these interactions with relevance to plasticity are outlined below.

The presence of GluN2A and GluN2B subunit-containing NMDARs at the synapse is stabilized by their interaction with the postsynaptic density (PSD) subfamily of membrane-associated guanylate kinases, which includes PSD-95, PSD-93, SAP102 and SAP97 [39]. Posttranslational modifications can alter how strongly GluN2A and GluN2B subunits bind to these PSD proteins, and this is one mechanism that regulates the availability of these subunits at the synapse: in turn this could influence the induction of plasticity. For example, Cdk2 phosphorylates GluN2B at Ser1480, which disrupts its interaction with PSD-95 and SAP102 and leads to a reduction in synaptic GluN2B [40,41]. Not only is this phosphorylation event unique to GluN2B [41], it is also regulated by synaptic activity [40]. Surface levels of GluN2B can also be regulated by phosphorylation at Tyr1472 by Fyn and Src; phosphorylation prevents the clathrin adaptor protein AP-2 binding to the GluN2B internalization motif YEKL, and thus inhibits endocytosis. Cdk5 is likely to be an additional component in this regulatory cascade, because inhibiting it encourages the interaction between Src and PSD-95, and consequently increases Tyr1472 phosphorylation [42]. Another example is the endocytosis of GluN2A, which is controlled by a dileucine motif at a different site (Leu1319 and Leu1320) [43], and thus its surface expression can be regulated independently. It is notable that GluN2B subunits also undergo more frequent endocytosis than GluN2A, and that these two subunits enter into different intracellular pathways, with GluN2B entering into recycling endosomes and GluN2A into late endosomes [43]. This suggests that activity- or neuromodulatory-dependent regulation of GluN2B may be a quicker or more sensitive way to alter the state of a synapse.

In addition to separate regulatory pathways to control the synaptic GluN2A and GluN2B levels, unique associations with different enzymes may also enable the subunits to contribute to opposing forms of plasticity. The most important example is probably the high affinity binding between the GluN2B CTD and the catalytic domain of Ca^2+^/calmodulin-dependent protein kinase II (CaMKII) [44]. There is a basal level of CaMKII present at the PSD bound to NMDARs [45], but this is supplemented by active, phosphorylated CaMKII that translocates to the PSD following the induction of LTP [46,47]. The interaction between CaMKII and GluN2B anchors CaMKII at the synapse in its active conformation [48]. LTP requires active CaMKII (reviewed in [49]), more specifically, the association between GluN2B and active CaMKII [50,51]. Therefore, the ability of GluN2B to mediate tight coupling between Ca^2+^ influx and CaMKII, and maintain extra active CaMKII in the vicinity of its substrates, such as AMPARs, to initiate the phosphorylation events that support synaptic strengthening, suggests that this subunit may play a crucial role in LTP. Another notable protein interaction at the postsynaptic spine is that between GluN2B and Ras-GRF1, a Ca^2+^/calmodulin-sensitive, Ras-specific guanosine diphosphate/guanosine triphosphate exchange factor that has been implicated in synaptic plasticity [52,53]. There are also proteins that associate indirectly with NMDARs but still retain a subunit preference, permitting an extra level of complexity in the modulation of NMDAR function by its subunit composition.

## 4. GluN2A and GluN2B NMDA receptor subunits in plasticity

As illustrated thus far, there are three main ways in which differential subunit composition could affect the type of plasticity induced. Firstly, the different channel properties conferred by these two subunits could encourage sufficiently distinct postsynaptic spine Ca^2+^ dynamics to trigger separate downstream plasticity pathways. Secondly, the unique associations made by the CTDs of GluN2A and GluN2B subunits provide a direct mechanism by which the subunits could couple to independent intracellular signalling cascades, and thus play different roles in synaptic plasticity. Finally, changes in the quantity or subcellular location of these subunits could alter the basal state of a spine, and thus affect the future induction of plasticity. Given these possibilities, it is interesting to ask to what extent experimental evidence supports the hypothesis that different NMDAR subunit compositions determine the outcome of a plasticity protocol.

### (a) Synaptic plasticity protocols

A wide variety of induction protocols can be used to induce synaptic changes in the hippocampus and the main paradigms are illustrated in table 1. There are considerable differences in the activity patterns used to induce even one direction of plasticity; therefore, it is possible that the synaptic changes induced by such diverse paradigms have distinct mechanistic underpinnings. It is important to consider this when delineating the NMDAR subunits involved in plasticity, as results are only truly comparable when the same protocol is applied.

**Table 1.** Different protocols used to induce LTP and LTD.

	spike pattern	response
LTP		
high-frequency stimulation (tetanus)	100 presynaptic stimulations at 100 Hz; this ‘tetanic’ train may be repeated up to four times to provide an even stronger paradigm, or to induce late-phase LTP	a post-tetanic potentiation is observed, generally considered to be a presynaptic phenomenon, followed by a transient NMDAR-dependent potentiation, known as short-term potentiation (STP) which then stabilizes to LTP
theta burst	consists of ‘bursts’ of three to five presynaptic stimuli at 100 Hz that are repeated at theta frequency (5 Hz) multiple times	a strong induction paradigm that generally produces an immediate and large increase in EPSP slope, pronounced STP and a high magnitude of LTP
pairing	combines approximately 100 low-frequency (2–5 Hz) presynaptic stimulations with a constant depolarizing postsynaptic current injection to hold the postsynaptic cell at a membrane potential of approximately 0 mV	the depolarization removes the Mg^2+^ block from the NMDARs and so maximizes Ca^2+^ influx. An immediate and large increase in EPSP slope results
spike timing	a single presynaptic spike is followed by a single postsynaptic spike within a limited time window (5–10 ms), repeated approximately 100 times at baseline acquisition frequency (0.1–0.3 Hz)	induces a slowly developing form of LTP, where EPSPs gradually increase and then stabilize. In the adult, a burst of postsynaptic spikes is required instead of a single spike to effectively induce LTP
LTD		
low-frequency stimulation	a high number of presynaptic stimulations (around 900) is given at 1–5 Hz	causes a large reduction in EPSP slope, followed by stabilization at a smaller magnitude of depression. Robust in young rodents; less reliable in adults
spike timing	a single postsynaptic spike is followed by a single presynaptic spike within a limited time window (5–20 ms), repeated approximately 100 times at baseline acquisition frequency (0.1–0.3 Hz)	induces a gradually developing form of LTD, where EPSPs gradually decrease and then stabilize. Reliable only in very young rodents

Various plasticity protocols have been used in combination with pharmacological and genetic manipulations to investigate the potential role of different NMDAR subunits in LTP and LTD. The first investigation into the roles of distinct NMDAR subpopulations in bidirectional plasticity compared the possible contributions of GluN2A/B and GluN2C/D subunits using broad-spectrum antagonists [54,55]. Subsequent studies have focused on dissecting whether differences exist between GluN2A and GluN2B, as these are the predominant subunits present at the juvenile and adult CA3-CA1 synapse. There are currently highly selective pharmacological tools to block GluN1/GluN2B receptors, primarily ifenprodil and its more potent derivative Ro 25-6981 [56]. Nevertheless, these are activity-dependent blockers, and the agonist concentration affects the extent to which these antagonists inhibit GluN2B subunit-containing NMDARs; for example, 3 µM Ro 25-6981 achieves 93% inhibition in the presence of 100 µM NMDA but only 62% in 10 µM NMDA [57]. The study of GluN2A is even more limited by the lack of selective antagonists for this subunit. NVP-AAM077 (hereafter referred to as NVP) is commonly used as a GluN2A-preferring antagonist, but it is now known to be less selective in rodents than had been assumed from recombinant human proteins and it actually shows only a 10-fold preference for GluN2A over GluN2B [56,58]. Therefore, data collected using NVP invariably have some block of GluN2B as well, but the magnitude of this block varies considerably with the concentration used.

Pharmacological and genetic manipulations allow the investigation of different aspects of NMDAR function. Pharmacological approaches are informative about the role of the NMDAR as an ion channel and do not directly address the importance of intracellular associations. They also will indiscriminately affect a particular NMDAR subtype on all cell types, for example interneurons and pyramidal neurons, where they may play different roles. In turn, this could have repercussions for the network that manifest at a circuit as well as at a behavioural level [59]. By contrast, constitutive and inducible genetic knock-outs provide information about the combined role of the NMDAR channel properties and synaptic presence, and can be directed to a specific cell type; however, given the changes in subtype composition throughout development, and even on the short timescales of metaplasticity, perturbing the balance of receptors is likely to cause other changes in the network or to recruit compensatory processes in the long term. In addition, pharmacological manipulations have often been performed in rats, whilst almost all the genetic manipulations discussed below have used mice, and species differences have not been excluded.

### (b) Long-term potentiation

An early study often cited to argue for the differential involvement of NMDAR subunits in the induction of LTP and LTD found that pharmacological block of GluN2A by NVP prevented tetanus- and pairing-induced LTP in three- to four-week rats but that antagonism of GluN2B by ifenprodil/Ro 25-6981 did not, but did instead block LTD induction [60]. Another study, using the same pharmacological concentrations and age of rats, also found that NVP completely blocked tetanus-induced LTP, though in this study ifenprodil and Ro 25-6981 partially blocked this form of LTP [61]. However, the data in both these studies were collected using a relatively high NVP concentration; thus, in addition to the aforementioned lack of NVP specificity, the resultant NMDAR-mediated current block was much greater with NVP (reduction of current: 53 ± 3.0% in [60] and 81 ± 3% in [61]) compared to the small reductions caused by Ro 25-6981 (36 ± 5% in [60] and 32 ± 3% in [61]). Therefore, this apparently selective role of the GluN2A subunit might instead arise from a threshold effect, whereby inhibiting more total NMDAR-mediated current could block LTP, especially given that a high level of Ca^2+^ influx is traditionally thought to be required for LTP induction. Indeed, when a study in mice controlled for this by selecting antagonist concentrations to reduce NMDAR-mediated current equally (40% reduction to match that possible by Ro 25-6981), pairing-induced LTP was not impaired irrespective of the antagonist used (NVP, Ro 25-6981 or the broad-spectrum NMDAR antagonist 2-amino-5-phosphonopentanoate (AP5)) [62]. The same antagonist concentrations, as titrated for their NMDAR current reduction in the aforementioned study, also did not impair LTP induced by a theta-burst paradigm in adult mice [63], though this low NVP concentration did partially block tetanus-induced LTP, while ifenprodil did not [64]. This suggests that tetanus-induced LTP is the most sensitive to GluN2A block. In addition to the extent of current reduction, the developmental stage is also likely to account for some of the different results reported. In a study using two-week-old rats, LTP was impaired by both GluN2A and GluN2B antagonism [65], probably because the developmental transition in subunit composition is not yet complete at this age in rats, as GluN2B antagonism reduced LTP in two-week-old rats but not in those over six weeks [66]. Some pharmacological studies have also been conducted *in vivo*, where, although it is more difficult to determine the exact concentrations that the CA3-CA1 synapse is exposed to, the variability introduced by different slicing angles, techniques and incubation solutions is avoided. Intrahippocampal infusions of Ro 25-6981 or NVP blocked tetanus-induced LTP *in vivo* in four- to six-week-old rats [67], whereas, when drugs were delivered intraperitoneally, only NVP blocked LTP with Ro 25-6981 having no effect [67,68]; this apparent discrepancy is likely to result from different levels of NMDAR block. Therefore, even *in vivo* studies have not yielded a clear conclusion.

As these pharmacological studies have provided little convincing evidence for a highly selective role of either subunit in LTP induction, an alternative hypothesis should be considered, whereby either subunit can support LTP provided they allow sufficient Ca^2+^ entry but the subunit that mediates most of the Ca^2+^ influx will have a greater importance. This subunit bias will be affected by neuronal activity patterns in behaving animals, whilst the induction protocol will influence the subunit bias in synaptic plasticity studies. The charge transfer modelling of Erreger *et al*. [34] suggests that the frequency of stimulation affects which subunit predominates; specifically, GluN2A should carry a majority of the current in tetanically induced LTP, whilst GluN2B might carry more current in lower frequency protocols [34]. Indeed, Ro 25-6981 blocked the induction of spike-timing-dependent LTP (t-LTP) in acute hippocampal slices from adult mice [69,70], which is induced by a low-frequency paradigm, whereas it did not impair tetanus-induced LTP [70]. However, complicating this interpretation, Zhang *et al*. [70] found, albeit with a high and thus less selective concentration, that NVP also blocked t-LTP. Furthermore, Gerkin *et al*. [71] found that an equivalent concentration of Ro 25-6981 did not block t-LTP in cultured hippocampal neurons, but NVP did; performing pre–post pairings at 1 Hz, rather than 0.1–0.2 Hz, and the use of cultured neurons, might account for this different result. Other studies have provided further evidence for a subunit bias owing to charge transfer. For example, a pairing protocol should limit the importance of subunit kinetics, as the postsynaptic neuron is held at a depolarized potential to remove the Mg^2+^ block. Therefore, neither subunit would be predicted to dominate, and any bias would rather be determined by the numbers of each subunit present at the synapse. Indeed, Berberich *et al*. [72] found that pairing-induced LTP was not blocked by NVP, Ro 25-6891 or AP5 when used at sufficiently low concentrations to have a minimal impact on charge transfer during induction; however, when antagonist concentrations were increased to a level that significantly reduced charge transfer, both GluN2A and GluN2B antagonists caused an equivalent reduction in the magnitude of LTP. It is also important to consider the complication introduced by the large triheteromeric population of NMDARs; a recent study found that only high concentrations of Ro 25-6981 could impair theta-burst-induced LTP, possibly because the antagonist starts inhibiting these triheteromers [9].

Overall, therefore, the conclusion from the current pharmacological data, with the caveat that no subunit-selective GluN2A antagonist exists, is that either subunit is capable of supporting LTP induction, provided they can mediate sufficient charge transfer (and associated Ca^2+^ influx). Which subunit mediates most charge transfer will be influenced by age, because the relative abundance of these two subunits changes over development [20,28,30]. Within a given age range, the induction protocol used may bias which subunit makes a greater contribution because of the different kinetics of the two subunits. Specifically, at the two extremes of frequency, t-LTP might have a greater contribution from GluN2B subunit-containing NMDARs, while tetanus-induced LTP could have a bigger contribution from GluN2A subunit-containing NMDARs [34]; the greater ability of GluN2A subunit-containing NMDARs to drive the induction of tetanic LTP has received additional support from modelling work [73]. The potential importance of stimulation frequency during induction means that, in order to fully explore the role of GluN2A and GluN2B in plasticity, it is vital to further investigate the types of activity patterns that drive synaptic changes in behaving animals.

However, pharmacological antagonists only block the ligand binding and channel properties of the NMDAR, and therefore pharmacological studies do not directly address whether the physical presence of NMDAR subunits at the synapse could play a role additional to that in mediating Ca^2+^ influx. The most important other function in plasticity is likely to be related to the direct and indirect intracellular associations made by the GluN2 CTD. Genetic manipulations that remove or alter the GluN2 subunits may shed some light on whether subunit selectivity in LTP induction could arise through these interactions; of particular interest is whether the GluN2B subunit plays a crucial role owing to its association with CaMKII.

A conditional genetic knock-out of GluN2B subunits in the CA3 subfield abolished tetanus-induced LTP at the commissural-CA3 and commissural/associational-CA3 synapses [74]. In mice lacking GluN2B in the CA1 and neocortex, LTP induced by two tetani was impaired, but this deficit could be overcome by giving multiple tetani [75]. A smaller effect on LTP was seen in another forebrain-specific GluN2B knock-out mouse, where LTP was deficient only when induced by a pairing protocol and not by a stronger protocol using two tetani [76]. Other genetic manipulations that indirectly affect the levels of GluN2B have also shown considerable LTP impairments. For example, kinesin-like protein KIF17 transports GluN2B subunit-containing NMDARs to the synapse, and KIF17 knock-out mice [77], or mice carrying mutations in KIF17 that disrupt its loading or unloading of GluN2B [78], show reduced synaptic GluN2B and also abolished (KIF17 knock-out) or impaired (KIF17 mutant) LTP induced by a single tetanus. However, when interpreting results from genetic manipulations as evidence for a role of GluN2B in LTP, it must be remembered that such long-term manipulations could trigger compensatory changes at the cellular level, such as increased recruitment of CaMKII by other PSD components, or even at the network level, through changes in inhibition, for example. Of particular note, GluN2B knock-out lines also show small reductions in GluN2A [77,78], GluN1 [74] or spine density [74,75]. Given that GluN2B subunits must therefore contribute to the regulation of NMDAR levels and postsynaptic structural integrity, it is difficult to attribute fully the LTP impairments found following chronic reduction of GluN2B to a unique structural/interaction role of the GluN2B CTD in LTP.

Therefore, it is interesting that shorter term manipulations, despite only reducing the GluN2B content, have produced stronger effects than complete genetic knockouts. For example, GluN2B knockdown with RNA interference (RNAi) caused a profound reduction in the magnitude of LTP induced in two-month-old rats even though a very strong LTP protocol (four tetani) was used [79]. LTP was also abolished in slices incubated for 2 h with a membrane-permeable GluN2B C-terminal peptide that disrupted the GluN2B–PSD interaction and caused a consequent decrease in synaptic GluN2B content [80]. Nevertheless, as mice with reduced synaptic GluN2A [81] or GluN2A knock-out [82] also exhibit reduced LTP, which can be recovered by a stronger induction protocol with multiple tetani [83], these experiments alone do not provide convincing evidence of a unique role of the intracellular interactions made by GluN2B.

The converse experiments, using subunit-selective overexpression, show that LTP is enhanced in adult [84] and aged [85] mice and adult rats [86] by increasing GluN2B. Cdk5 knock-out mice with elevated levels of synaptic GluN2B also show enhanced LTP [87]. By contrast, GluN2A-overexpressing mice do not show increased LTP [88], suggesting that GluN2B may play a unique role. However, elevated LTP was observed in dysbindin knockout mice that had enhanced GluN2A-mediated currents [89]; thus, it is not completely clear whether the enhanced LTP in GluN2B-overexpressing rodents arises from higher levels of Ca^2+^ influx owing to the increased NMDAR-mediated current or because of an important contribution of the GluN2B subunit itself. Therefore, data from knock-out and overexpression studies suggest that the GluN2B subunit is important for the induction of LTP, but do not convincingly distinguish between a unique role of GluN2B at the synapse or a general threshold effect. In the latter case, the reduction or increase in synaptic NMDAR content produced by these genetic manipulations would alter the magnitude of LTP simply because of non-specific changes in the amount of Ca^2+^ influx during induction.

Rather than attempt to demonstrate a unique role for GluN2B by excluding this threshold possibility, studies have instead focused on manipulating the distinguishing features of the GluN2B subunit, in particular its intracellular interactions. The most convincing evidence for a uniquely important role for the interaction between GluN2B and CaMKII in LTP comes from experiments directly perturbing this association. The first data came from work by Barria & Malinow [50]; they showed that LTP was blocked in organotypic hippocampal slices transfected with a GluN2B construct impaired in binding to CaMKII. Moreover, the LTP impairment caused by transfecting recombinant GluN2A, which drives a switch in synaptic NMDAR content from GluN2B to GluN2A, could be ameliorated by transfecting a mutated form of GluN2A with an enhanced ability to bind CaMKII [50]. This further suggests that an important function of wild-type GluN2B is to maintain sufficient CaMKII at the synapse. These findings have since been extended to acute slices; an inducible mutation that weakens the GluN2B–CaMKII interaction impaired LTP induced by both a tetanic and 10 Hz protocol [51], while a knock-in mouse with two point mutations that decrease the GluN2B–CaMKII interaction also showed a large reduction in two-tetani-induced LTP [90]. This interaction is regulated by both components as inhibiting CaMKII, which initially disrupts the GluN2B–CaMKII association, leads to downregulation of synaptic GluN2B content within 2 h and a concomitant impairment in LTP [80]. Inhibition of CaMKII was removed, and hence CaMKII kinase activity restored, before LTP was induced, and therefore the LTP deficit observed by Gardoni *et al.* [80] could be attributed to an altered synaptic subunit composition.

One reason why the GluN2B–CaMKII interaction is so important for LTP might be its effect on AMPARs. Phosphorylation of Ser831 on GluA1 may be necessary for AMPAR insertion, and this phosphorylation does not occur when the GluN2B–CaMKII interaction is blocked [90], which may hinder synaptic strengthening. Thus, through its strong affinity for CaMKII, it does seem that GluN2B may play a special role at the synapse additional to its function as an ion channel. This was particularly clear when, at an age when pharmacological inhibition of GluN2B with Ro 25-6981 no longer blocked pairing-induced LTP, LTP was impaired by RNAi knock-down of GluN2B [91]. This difference seems directly related to a unique role of the GluN2B CTD as, following RNAi knock-down of GluN2B and the resultant LTP impairment, LTP could be restored by an RNAi-resistant GluN2B or a chimaera of GluN2A with the GluN2B tail, but not a chimaera of GluN2B with the GluN2A tail [91]. Thus, the physical presence of GluN2B may be important irrespective of its contribution to Ca^2+^ influx. This explains how GluN2B could have a unique and fundamental role in LTP induction, despite pharmacological block of GluN2B not impairing the LTP induced by certain paradigms.

However, this does not mean that the GluN2A CTD makes no contribution to LTP. It has been shown that GluN2A subunit-containing NMDARs alone can trigger a form of LTP dependent on the Ras-GRF2/Erk Map Kinase pathway [52], suggesting that unique interactions may occur. Indeed, a mouse line with a GluN2A C-terminal truncation showed impaired tetanus-induced LTP, with the remaining LTP supported by GluN2B-subunit-containing NMDARs [66,92]. However, as the truncation mutation also caused an overall reduction in GluN2A levels in this mouse line, as well as a possible switch to a pure triheteromeric NMDAR population, it is possible that the LTP impairment arose from a threshold effect from reduced Ca^2+^ influx rather than a unique role of the GluN2A CTD. Supporting the threshold interpretation, stronger induction protocols expected to promote higher levels of Ca^2+^ influx could overcome the LTP deficits in mice with a GluN2A C-terminal truncation both in this [66] and another study [93]. Thus, the evidence for a privileged role of GluN2A in LTP induction is weaker.

One vital consideration when investigating a selective role of NMDAR subunits in LTP is that CA3-CA1 synapses are not a uniform population. Changes in the extracellular field or whole-cell response to a plasticity protocol are, therefore, the summed changes from a heterogeneous population of synapses that may respond differently to the same activity pattern. Structural imaging and molecular labelling studies have revealed that, even in the adult brain, there are different categories of postsynaptic spine shape that also have distinct receptor signatures [94]. Imaging of individual spines has shown that GluN2B antagonists selectively reduce glutamate uncaging-evoked EPSCs and Ca^2+^ transients in small spines [38] indicating that GluN2B is concentrated in such spines. This is of particular interest because chronic imaging experiments have revealed that, whilst the volume of all spines increases following an LTP induction protocol, this expansion is only preserved in spines initially classified as small, but is transient in others [95]. This suggests that these distinct types of spines, with their different NMDAR subunit compositions, may engage differently in plasticity processes, and thus could play independent roles even in the mature brain. Therefore, using the variability in synapse structure and composition may be a fruitful additional approach to study the role of NMDAR subunits in LTP and information processing in general. Electron microscopy and immunolabelling have revealed that these different spine populations are asymmetrically distributed in the adult mouse brain; specifically, postsynaptic CA1 spines receiving input from the left CA3 are mainly small and rich in GluN2B subunit-containing NMDARs, whereas those receiving input from the right CA3 tend to be larger and richer in AMPARs [94,96]. This enabled these different types of spine, with their different GluN2B content, to be targeted optogenetically to test the hypothesis that GluN2B subunits are particularly important for LTP induction. By injecting channelrhodopsin-2 (ChR2) under the control of a Cre-dependent promoter into either the left or right CA3 of CaMKII-Cre mice, the left or right excitatory CA3 input onto CA1 could be selectively recruited. It was found that only left CA3-CA1 synapses showed LTP induced by spike timing-dependent or theta-based protocols [69]. Moreover, this was likely a result of the higher GluN2B content of spines receiving left CA3 projections, as Ro 25-6981 caused a greater reduction in NMDAR-mediated current in left-injected mice and also blocked the observed plasticity [69]. Although some GluN2B was present at the right CA3-CA1 synapses, they appeared incapable of expressing LTP; an explanation is that they are already at the maximum possible synaptic strength that can be maintained. It will be interesting to test whether a similar asymmetry is found following stronger induction paradigms such as tetanus-induced LTP, though current limitations of ChR2 kinetics in driving pyramidal neurons at a sufficiently high frequency preclude direct testing of this.

In summary (figure 1*b*), the most parsimonious conclusion from the available evidence is that both GluN2A and GluN2B subunits can support the requisite Ca^2+^ influx to induce LTP. However, which subunit predominates in mediating this Ca^2+^ influx, and thus contributes more to LTP induction, depends on the protocol and also developmental stage, as the latter affects subunit abundance at the postsynaptic spine. Provided the NMDARs present can support sufficient Ca^2+^ influx, the GluN2B subunit seems to play a unique additional role because of its association with CaMKII. This interaction targets CaMKII to the vicinity of Ca^2+^ influx at the PSD and also provides a binding site for the additional activated CaMKII recruited following an LTP induction protocol. Finally, GluN2B helps maintain CaMKII activation and localize it at the PSD in the vicinity of its cellular substrates enabling it to trigger the downstream pathways that mediate the expression of LTP.

### (c) Long-term depression

Given the possible bias towards an important contribution of the GluN2B subunit to LTP induction, an opposite bias might have been expected for NMDAR-dependent LTD. However, studies into the NMDAR subunit dependence of LTD have produced even more contradictory results than those for LTP, making it hard to draw unifying conclusions. The different types of findings are illustrated below and possible explanations for the controversies are offered. One major complication in this field is the strong dependence of LTD induction on developmental age, because small changes in age affect the magnitude or even presence of LTD [97]. Nevertheless, this may provide information about the mechanisms involved, especially given the changing pattern of subunit expression across development [20,30]. Very few studies have addressed subunit involvement in spike timing-dependent LTD in the hippocampus, and therefore the following discussion will focus on low-frequency-induced LTD. Nevertheless, groups investigating this form of LTD have often drawn opposing conclusions about subunit-specificity, despite using very similar experimental preparations. For example, some pharmacology-based studies have found that a concentration of NVP that blocked LTP did not impair LTD in culture [71], in acute slices [60] or *in vivo* [68], suggesting that GluN2A subunits are not required for LTD induction. By contrast, other studies have found that NVP concentrations that impaired LTP also blocked LTD [61,65]. It is possible that NVP causes a substantial reduction in NMDAR-mediated current that prevents LTP and, depending on the precise concentrations used, sufficient Ca^2+^ influx remains to trigger the downstream molecular cascades that mediate LTD. The importance of antagonist concentration in determining experimental conclusions is illustrated by the findings of Fox *et al*. [67], where, of two NVP concentrations administered intraperitoneally that blocked LTP *in vivo*, only the higher concentration blocked LTD. Overall, therefore, the extent to which GluN2A subunits are engaged in LTD induction under physiological circumstances is not yet clear, but there certainly is no pharmacological evidence that they play a mandatory or unique role.

There has been a particular focus on a possible role of GluN2B subunit-containing NMDARs in LTD. Some groups find that the GluN2B-specific antagonists Ro 25-6981 and ifenprodil block LTD in acute slices [60,64], in culture [71] and *in vivo* [67,68], even when a larger NMDAR-mediated current reduction by NVP has no effect [60,68,71]. However, as with the contradictory findings reported with GluN2A antagonism, many other studies have found that the same manipulation has no effect on LTD induction in acute slices, despite using equivalent or higher concentrations of GluN2B antagonists [61,65,98,99]. The slice orientation used may partially explain the discordance between these findings, since Bartlett *et al*. [100] found that coronal slices (as used in [60]) had a form of LTD that was sensitive to Ro 25-6981, whereas sagittal slices (as used in [65]) had a GluN2B-independent form of LTD, likely owing to the stronger preservation of cholinergic projections with a sagittal cutting angle. Indeed, when they blocked muscarinic acetylcholine receptors, it unmasked a GluN2B-dependent LTD [100]. The contradictory results found in slices cut in the transverse angle, where studies have found either GluN2B-dependence [53,64] or GluN2B-independence [98,99] of LTD, might result from subtle variations in the integrity of cholinergic fibres.

One further factor contributing to the variability in the sensitivity of LTD to GluN2B antagonism may be the extent to which extrasynaptic GluN2B subunit-containing NMDARs are recruited. The traditional low-frequency induction protocol is sometimes combined with application of the glutamate-uptake inhibitor *threo*-β-hydroxyaspartate (TBOA), which would likely increase activation of the extrasynaptic receptor population. Contrasting effects have been reported following TBOA application: it has not affected GluN2B involvement in LTD [65], it has produced a GluN2B-dependent LTD [101], or it has even decreased the magnitude of LTD [98]. The last finding suggests that extrasynaptic, possibly GluN2B subunit-containing, NMDARs could exert a negative modulatory role on LTD under some experimental conditions. This suggestion is supported by a study finding that ifenprodil block of GluN2B actually enhanced the magnitude of LTD [102]. However, it cannot be excluded that the discrepancy between these findings results from the complex mode of action of the non-competitive GluN2B antagonists used, as the extent of their block varies with agonist concentration [57], and such concentrations are likely more variable when glutamate uptake is blocked. Overall, therefore, the contradictory findings mean the precise nature of the role played by GluN2B subunit-containing NMDARs in LTD is anything but clear, and the induction of LTD may be particularly sensitive to experimental conditions.

Transgenic approaches have also not provided conclusive support for a unique role of either subunit in LTD and, in fact, most of the findings have been negative. A reduction in synaptic GluN2B levels, achieved by disrupting the association of GluN2B with PSD-95 in an acute manner, did not alter LTD, despite being sufficient to impair LTP [80]. The GluN2A knock-out mouse also showed no LTD impairment, though the same study also found no reduction in LTP [103]. Furthermore, overexpression of GluN2B did not affect the magnitude of traditional 1 Hz stimulation-induced LTD [86], and neither did direct [88] or indirect [89] GluN2A overexpression, although GluN2A overexpression did reduce LTD induced by 3 or 5 Hz stimulation without affecting LTP [88]. The only clear positive findings have been that GluN2B knock-out mice [75] and KIF17 knock-out mice, which have a consequent reduction in synaptic GluN2B [77], both have impaired LTD. However, this is not evidence for a subunit-selective role of GluN2B in LTD because these manipulations also caused reduced LTP. Overall, there is no clear evidence from transgenic approaches that either subunit plays an irreplaceable role in LTD induction, though impairments have been more frequently associated with GluN2B-specific manipulations.

A final possibility is that one subunit makes a more important contribution to LTD through its unique intracellular interactions. This may be because it facilitates coupling of Ca^2+^ influx to the downstream pathways that mediate a reduction in synaptic strength. Alternatively, NMDARs could play a different role in LTD, where their activation is still required to trigger downstream signalling cascades, but not to mediate Ca^2+^ influx. Evidence for this latter proposal is the demonstration that NMDAR-dependent LTD could still be induced despite NMDAR-mediated ion flux being blocked [104]. This suggests that the basal level of Ca^2+^, rather than Ca^2+^ influx through NMDARs, is necessary for LTD induction and may explain why no clear findings have emerged from studies using subunit-specific pharmacological antagonists. It also implies that any subunit-selective function of NMDARs would emerge solely through their unique intracellular associations. As predicted by the requirement for CaMKII in LTP induction alone, genetic disruption of the GluN2B–CaMKII association did not impair LTD [51]. However, other interactions have been identified that could be important. For example, LTD alone was impaired in mice with a genetic knock-out of the p75 neurotrophin receptor [105]. The specific mechanism responsible for this deficit was reported to be that pro-brain-derived neurotrophic factor could not activate the p75 neurotrophin receptor to enable LTD and, because the mouse line also had an overall reduction in GluN2B levels, it suggests a possible signalling role of GluN2B. Another important association might be that between Ras-GRF1 and the GluN2B CTD, as LTD is impaired in Ras-GRF1 knock-out mice [53]. The level of Ras-GRF1 may determine the type of depression induced under physiological conditions, as LTD is GluN2B-dependent when this association predominates [100]. Furthermore, acetylcholine concentration is inversely correlated with the level of the GluN2B–Ras-GRF1 interaction [100], suggesting that activity of the cholinergic system could modulate the subunit dependence of LTD induction.

In summary, the data are inconclusive, but neither the GluN2A nor the GluN2B subunit seems to play an obligatory role in the induction of LTD. Nevertheless, the GluN2B subunit has been implicated more frequently, perhaps because of its unique associations with components of pathways that modulate or mediate LTD.

## 5. Metaplasticity

The basal state of the synapse is likely to play a crucial role in determining the nature of its changes in response to a particular input. It has even been suggested that it affects whether the LTP expression mechanism is pre- or postsynaptic [24]. Given that there is no convincing evidence for complete subunit selectivity in the induction of either direction of plasticity, it is conceivable that GluN2A and GluN2B subunits play a subtler role, biasing the synapse towards the induction of either LTP or LTD. Thus, it has been investigated whether altering the relative or absolute levels of either subunit changes the basal state of a synapse sufficiently to influence the future induction of plasticity.

Activity-dependent changes in the patterns of subunit expression take place in the forebrain during development [20,30], and even a short history of neuronal activity can change the subunit balance at the CA3-CA1 synapse in young rodents [106–108]. To investigate whether changes in subunit composition alter the response of the synapse to further activity, Xu *et al*. [109] used 600 low-intensity pulses at varying frequencies to ‘prime’ the synapse; this priming was insufficient to change synaptic weights but did alter the GluN2A/GluN2B ratio. Following this priming, different plasticity protocols were applied. The magnitude of LTP was enhanced, and LTD reduced, by low-frequency priming that decreased the GluN2A/GluN2B ratio. By contrast, an elevated LTD and suppressed LTP was seen if the synapse had been primed with high-frequency stimulation that increased the GluN2A/GluN2B ratio. They also used a plasticity protocol that did not change synaptic weights in a ‘naive’ slice and found that this threshold protocol could induce LTP if preceded by low-frequency priming, but induced LTD if preceded by high-frequency priming. Moreover, the effect of priming on the outcome of this threshold protocol could be replicated by direct manipulation of the GluN2A/GluN2B ratio using partial block by pharmacological antagonists (having titrated concentrations with AP5 so that the NMDAR-mediated current reduction itself could not explain the effect) [109]. This result suggests that synaptic activity that increases the relative GluN2B content of a synapse biases it towards LTP, whereas a relative increase in GluN2A encourages the induction of LTD. The effect of prior activity has also been measured at the single synapse level [110]. Sparse transfection of neuronal cultures with a construct that blocks presynaptic vesicle release was used to silence some inputs; size-matched postsynaptic spines, one with a silenced input and the other with an active input, were then exposed to a glutamate uncaging-based LTP induction protocol. The silenced synapses showed LTP and ensuing spine growth with a low-intensity protocol, whereas a higher number and longer duration of glutamate uncaging events were required to induce LTP and structural changes in spines that had been receiving an active input. Moreover, GluN2B appeared to mediate the increased propensity for potentiation, as the GluN2B-mediated current was enhanced at silenced synapses [110]. Therefore, both these studies suggest that metaplastic changes that cause a relative or absolute increase in the synaptic GluN2B content will bias a synapse towards LTP.

Neuromodulatory factors are known to influence how a synapse responds to a given activity pattern, and it is possible that they alter the basal state of the synapse in a subunit-selective manner. Dopamine levels modulate plasticity; for example, dopamine application augmented tetanus-induced LTP via a cascade involving the D_1/5_ receptor, PKA and Src family kinases [111]. This LTP enhancement was blocked by Ro 25-6981 [111], suggesting that an increase in GluN2B subunit-containing NMDARs may be the expression mechanism for dopamine-dependent metaplasticity, which is in line with the findings from activity-based metaplasticity studies. However, not all metaplasticity studies have supported the conclusion that GluN2B encourages LTP. Priming by activation of G protein-coupled receptors and their downstream actuators in the Src family of kinases showed that metaplastic changes increasing GluN2B had the opposite effect on plasticity to the aforementioned studies [112]. Instead, Src kinase (activated by the pituitary adenylate cyclase-activating peptide 1 receptor; PAC1R) was shown to phosphorylate GluN2A, and thus enhance GluN2A-mediated currents, whereas Fyn kinase (activated by the D_1/5_ receptor) phosphorylated GluN2B to enhance GluN2B-mediated currents. In turn, this influenced the response to a series of different frequency induction protocols: the switch from LTD to LTP induction occurred at 10–20 Hz without prior drug treatment, but exposure to PAC1R-activating drugs and a consequent GluN2A enhancement meant that LTP was induced at lower frequencies; by contrast, the LTP induction threshold was shifted to higher frequencies if the D_1/5_ receptor had been activated, and hence GluN2B was increased [112]. This, therefore, suggested that GluN2A promotes LTP induction. Overall, further investigation into metaplasticity may help unravel how neuronal activity can alter synaptic weights and whether there is a contribution from changes in NMDAR subunit composition to synaptic priming effects. This is especially important given that learning and memory does not take place in an isolated fashion but against a background of prior neuronal activity and neuromodulatory input.

## 6. NMDA receptor subunits in behaviour

Synaptic plasticity is widely considered a cellular model for learning and memory [3,113], and there were early indications of an important role for NMDARs in certain forms of learning and memory [4,5]. In parallel to investigations into whether there is a subunit-selective contribution to LTP and LTD, subunit-specific manipulations have also been used to interrogate whether GluN2A and GluN2B subunits are important for behavioural tasks with different cognitive demands. In some cases, impairments in one direction of plasticity could be correlated with performance deficits.

GluN2A knock-out mice were the first to be studied behaviourally. The initial characterization of these mice suggested relatively pervasive memory deficits compared with C57BL/6 controls [82], but this was later attributed to the high degree of CBA strain character remaining in their genetic background, a line known to perform worse on the Morris water maze (MWM) task [114]. Once on an almost pure C57 background, the behavioural deficits found were relatively limited: GluN2A knock-out mice and mice lacking the GluN2A CTD showed no evidence of long-term memory deficits, as mice were not impaired on spatial tasks acquired over a number of days (the MWM and radial arm maze) [115]. Mice with a knock-out of Neuropilin and tolloid-like protein 1 (Neto1), a component of the NMDAR protein complex, have an approximately one-third reduction in GluN2A at the PSD and an LTP impairment, but also acquired the MWM at a normal rate [81]. Furthermore, pharmacological manipulations have produced a similar result; Ge *et al*. [68] used the GluN2A subunit-preferring antagonist NVP injected intraperitoneally at a concentration that impaired LTP *in vivo*, and saw no deficits of NVP-treated rats either in MWM acquisition or consolidation. Thus, GluN2A does not seem necessary for incrementally acquired long-term memory tasks.

However, GluN2A manipulations have been associated with short-term spatial memory deficits, because GluN2A knock-out mice and mice lacking the GluN2A CTD were impaired on a non-matching-to-place T-maze task and also in their ability to distinguish between arms based on how recently they had been visited within a trial on the radial arm maze [115]. In further support of a role for GluN2A in short-term spatial memory, infusion of NVP into the CA1 impaired performance on a delayed alternation T-maze task [116]. It also seems that GluN2A subunit-containing NMDARs may contribute to the rapid formation of context or object representations. GluN2A knock-out mice, which had an increased threshold for LTP induction, were only impaired in a hippocampus-dependent contextual fear-conditioning paradigm when the task demands were increased by reducing context exposure before shock delivery [83], suggesting a deficit in the quick formation of a context representation. Neto1 knock-out mice were impaired in a displaced object recognition task, but not in a novel object recognition task [81]; both these tasks require relatively rapid learning about an object, but only the displacement task has a clear spatial component and clear hippocampal involvement (but also see [117]). Overall, mice with no or reduced GluN2A do show some learning impairments, which are limited to short-term memory and the rapid acquisition of spatial information. In order to test whether this is a particular role of GluN2A or a non-specific effect of reduced NMDAR-mediated currents (especially given the lack of NVP selectivity), these results should be compared to those from mice with equivalent genetic and pharmacological manipulations of GluN2B levels.

There is considerable evidence that pharmacological and genetic manipulations of the GluN2B subunit produce similar impairments to those described above, suggesting that the short-term memory deficits observed in GluN2A-deficient mice are primarily owing to a general reduction in NMDAR-mediated current. For example, mice with postnatal GluN2B knock-out in pyramidal neurons of CA1 and the dentate gyrus (DG) of the hippocampus showed very similar impairments to global GluN2A knock-out mice, namely a short-term spatial memory deficit in spontaneous alternation in the T-maze but normal performance in the MWM [76]. This finding is supported by pharmacological data from rats, where infusion of Ro 25-6981 or ifenprodil into the CA1 region of the hippocampus impaired performance in tasks requiring short-term spatial memory, though long-term memory was not tested [116]. Rats were impaired on a delayed alternation T-maze task with 5- and 30-s delays, but they only made significantly more win-shift errors than controls after a 30-s delay. Rats also had longer escape latencies on trial 2 of a delayed matching-to-place water maze task, but only when the retention interval was 10 min, not 30 s [116]. These two findings suggest that GluN2B subunits may become more important when delay times increase. It should be noted, though, that pharmacological antagonism of NMDARs will also affect interneurons [59]. In general, the similar short-term memory impairments seen following manipulations that remove or reduce either the GluN2A or GluN2B subunit suggest that this phenotype is primarily owing to a decrease in NMDAR-mediated currents rather than a selective contribution of either subunit.

Nevertheless, there is evidence that GluN2B subunits could play a role additional to that shared with GluN2A in short-term memory, because other studies have found more pervasive impairments following GluN2B disruption. For example, KIF17 knock-out mice, which have reduced synaptic GluN2B levels owing to a transport impairment, performed worse on the MWM task [77,78], a novel object test with long delays [77] and contextual fear-conditioning [77]. A CA1 and neocortical GluN2B knock-out mouse line was deficient in MWM acquisition, fear-conditioning and T-maze spontaneous alternation [75]. However, these impairments might be attributable to extra-hippocampal deficits [76]. Therefore, it is reassuring that a more acute manipulation involving RNAi-mediated knock-down of GluN2B in the hippocampi of rats also showed slower acquisition of the MWM task [79]. The authors also found a correlation between GluN2B level and MWM performance within an aged population of rats [79]. However, the nature of the GluN2B involvement cannot be correlated with one direction of plasticity as RNAi caused an LTP deficit but LTD was not investigated, while both the aforementioned genetically altered mouse lines had deficits in both LTP and LTD. Nevertheless, these studies suggest that GluN2B may play a role in long-term memory that was not seen in mice with GluN2A knock-out or reduction, though additional studies using acute manipulations restricted to the hippocampus are required to verify this conclusion.

To dissect further a possibly unique role of GluN2B in learning and memory, it is useful to investigate the behavioural deficits associated with a selective impairment of one direction of plasticity. For LTP, a selective impairment arises if the association between CaMKII and GluN2B is weakened. This manipulation has been found to cause deficits in acquisition of both the MWM task, which could be overcome by extended training, and also in a delayed spatial win-shift eight-arm radial maze task, where mice had to remember the baited arms from the first session, and then avoid them in the next trial to find the food reward [51]. The authors ascribed this deficit to an impairment in forming a spatial map. Another mouse line with genetic disruption of the CaMKII–GluN2B interaction engineered in a different way showed fewer behavioural impairments, being normal in MWM acquisition and probe trial performance at 1–2 h post-training, but, interestingly, did exhibit long-term consolidation deficits, as they performed worse in a probe test 24 h after training [90].

Furthermore, in contrast to the impairments seen following GluN2A overexpression [88], GluN2B overexpression has been shown to improve performance of adult [84] and aged mice [85] and rats [86] in a battery of tests including context and cued fear-conditioning, object recognition memory at longer retention intervals, MWM acquisition and spatial short-term memory. Cdk5 knock-out mice, which have increased synaptic GluN2B owing to a reduction in its degradation were also found to have improved contextual learning in a context-dependent fear-conditioning paradigm [87]. All these behavioural improvements were associated with increased LTP, while LTD, when investigated, was found not to change [86]. Stronger evidence for a link between subunit-specificity in LTP and learning comes from tasks where learning and postsynaptic responses can be measured simultaneously. Using the trace-conditioning paradigm in rats, Valenzuela-Harrington *et al*. [118] showed that acquisition of this task was associated with an increased strength of the medial perforant pathway-DG synapse of the hippocampus. Moreover, systemic administration of Ro 25-6981 blocked both task acquisition and the changes in synaptic strength. Therefore, possibly through its contribution to LTP induction via CaMKII, GluN2B seems to have an important role in learning and memory.

Some behavioural deficits have also been related to a GluN2B-dependent LTD impairment. Ge *et al*. [68] administered Ro 25-6981 intraperitoneally to rats, which impaired LTD but not LTP *in vivo*, and found that, while acquisition of a MWM task was not impaired by this manipulation, rats that received Ro 25-6981 on the training day could not recall the platform location 24 h later owing to a consolidation-related deficit. This compromised performance could be mimicked by injection of a peptide that prevented AMPAR endocytosis (Tat-GluA2_3Y_), suggesting that the impairment was related to an LTD-like process. However, an alternative interpretation is that this manipulation disrupted homoeostatic resetting of the network and that this process is required for consolidation.

A finding reported by a number of groups is a possible link between LTD and reversal learning. Duffy *et al*. [101] found that subcutaneous administration Ro 25-6981 blocked LTD and impaired reversal learning in mice, and deficient reversal learning in the MWM was also found in mice with GluN2B knock-out in the hippocampus, though LTD was not tested [76]. In rats, GluN2B-dependent LTD could not be induced in naive animals *in vivo*, but could be induced once animals experienced MWM training; when rats that had previously acquired the MWM were tested on a reversal phase, those rats treated with either Ro 25-6981 or the Tat-GluA2_3Y_ peptide were impaired in reversal learning [119]. In further support of a role of GluN2B-dependent LTD in reversal learning, enhancing LTD by subcutaneous administration of d-serine increased the rate at which the mice learnt the reversal phase in the MWM [101], while a mouse line with enhanced GluN2B-mediated currents showed no improvement in the acquisition of an MWM task, but did show faster reversal learning [87]. Thus, many of the studies investigating a role of NMDAR subunits in behaviour have found a ‘perseverance phenotype’ that correlates with an impairment in GluN2B-dependent LTD.

There is a large body of evidence to suggest that synaptic plasticity supports learning and memory [4,5,118,120], but the link is not yet resolved [121,122]. Therefore, studies that correlate plasticity and behaviour have been informative, irrespective of whether the underlying, possibly subunit-selective, mechanisms are known. Nevertheless, correlations between plasticity deficits and learning and memory impairments do not provide a causative link. Moreover, we should be cautious in concluding that such studies provide definitive evidence given the possible compensatory mechanisms and network changes that may be triggered by the experimental manipulations described here. Of course, even if plasticity does support learning and memory, we still do not know what types of plasticity would be engaged in tasks with different cognitive demands, and thus, with all this considered, it is no surprise that the findings from these studies are complex and often contradictory. In some cases, correlations have been found between an impairment in one direction of plasticity and task performance, and occasionally particular subunits implicated. However, equally, some manipulations that caused large impairments in the magnitude of plasticity had little or no effect on the behavioural tests chosen, only affected certain aspects of the tasks, or even only caused an impairment when task difficulty was increased or demands were changed, such as by introducing a delay. The aforementioned hemispheric asymmetry in the distribution of the GluN2B subunit and LTP in the mouse CA3-CA1 synapse may help further elucidate the role of synaptic plasticity in learning and memory, and also determine if there is a particular subunit involvement. Although it is not known how this asymmetry arises in development and is maintained during adulthood, evidence that it may be important in memory processing comes from *inversus vicerum* mice that show disrupted asymmetry and display memory impairments [123].

## 7. Summary

The hypothesis that different GluN2 subunits selectively mediate different directions of plasticity is not supported by the available studies. Although the lack of specific GluN2A antagonists prohibits the conclusive testing of this hypothesis, when manipulations control for the magnitude of NMDAR-mediated current reduction, there is no clear evidence, as yet, that either subunit plays an irreplaceable role in LTP induction. Nevertheless, the different kinetics conferred on the NMDAR by the GluN2A and GluN2B subunits means the contribution of each subunit to charge transfer may vary according to the pattern of presynaptic activity, and therefore they may not play an equal role in potentiation under physiological conditions.

Irrespective of the induction paradigm used, however, most evidence suggests that the GluN2B subunit has a greater importance for LTP induction. An explanation for why GluN2B can exert a strong influence on LTP in the adult hippocampus, despite comprising a smaller proportion of GluN2 subunits than early in development, would be if GluN2B subunit-containing NMDARs carry more Ca^2+^ influx per unit of current [38]. An alternative explanation, which is more in line with the prevailing evidence, is that GluN2B is particularly important for LTP induction because its presence at the spine anchors CaMKII at the PSD. Thus, GluN2B subunit-containing NMDARs enable activation of the downstream signalling cascades that mediate synaptic strengthening, irrespective of whether they support a majority of the Ca^2+^ influx. This suggests that GluN2B is a necessary factor for LTP induction under naturalistic conditions, but, importantly, whether or not it is sufficient to support the requisite Ca^2+^ influx may depend on the pattern of presynaptic activity. Moreover, although spine-based imaging studies and asymmetry studies have suggested that small spines, which tend to be GluN2B-rich, have a greater propensity for the induction and maintenance of LTP, the presence of GluN2B may not be sufficient if downstream signalling pathways and structural changes are already saturated.

The LTD literature is ambiguous, although a greater number of studies and the developmental profile of LTD would support a more central role of GluN2B in its induction. Exactly why this may be is not clear, but could relate to intracellular associations distinct from that with CaMKII. Most plasticity studies investigate the response to induction protocols at either the cellular or extracellular field potential level, and thus record the summed changes at many synapses. However, synapses are not equal, and instead have different structural and molecular signatures, and this may help explain at least some of the contradictory findings in the plasticity literature. Overall, given that GluN2B seems important for both LTP and LTD, a parsimonious hypothesis is that this subunit confers the synapse with malleable properties.

Given the contradictory conclusions of plasticity studies, it is unsurprising that neither subunit has yet been found to have a clear role in learning and memory. What the current literature does suggest is that both GluN2A and GluN2B support short-term memory processes. There is also some evidence that GluN2B subunit-containing NMDARs may make a greater contribution to learning when information must be retained for a longer delay or there is incremental task acquisition across a number of days.

It may seem surprising that short-term memory tasks often appear the most susceptible to genetic lesions or pharmacological block of NMDARs, especially in light of the proposal that NMDAR-dependent LTP supports task acquisition through incremental learning [124]. However, a number of factors make it premature to conclude that this necessitates a revision of the theory linking plasticity and learning. Firstly, NMDARs have roles additional to their involvement in plasticity, as further described later. Secondly, a global knock-out would also remove NMDARs located on interneurons, and hence may alter the network dynamics important for short-term memory [125]. Thirdly, in the few studies where the subunit knock-out is restricted to pyramidal cells in the hippocampus, navigation systems may still be affected, and how that could interact with the cognitive demands of short- and long-term memory tasks is not known. Fourthly, even localized manipulations have been chronic, and so compensatory mechanisms are likely to be recruited; it is conceivable that short-term memory requires greater online processing power or rapid, large-scale network changes, either of which might need a higher level of Ca^2+^ influx to drive them, and thus would be more sensitive to manipulations that reduce NMDARs and hence total Ca^2+^ influx. Finally, the fact that these manipulations also often impair LTP does not necessarily imply a link between LTP and short-term memory, it only shows that both processes rely on NMDARs. Indeed, short-term potentiation also requires NMDARs and may have subunit preference [9].

In some studies, it has been possible to relate a behavioural impairment to a selective deficit in one direction of plasticity. In particular, there is evidence that the gradual acquisition and consolidation of information may be supported by the GluN2B–CaMKII interaction, which is also important for LTP. Another common finding is that the mechanisms that support reversal learning may involve a GluN2B-dependent LTD-like process. One caveat of studies relating learning impairments with LTD deficits is that many laboratories find that they cannot induce LTD in adult rodents, despite rodents of that age still being able to perform the associated memory tasks. This suggests that either the low-frequency paradigms used to induce LTD do not successfully mimic the type of activity that triggers reduction of synaptic weights *in vivo*, or, even, that synapse-specific reductions in strength do not underlie learning; instead, they may be more important for circuit refinement during development. One way to reconcile these observations would be that synaptic strengthening always supports the acquisition of new information, but a concomitant depression is also necessary in behavioural tasks that require re-learning (reversal learning, extinction) or refinement of learning (recognition memory) to increase the gain of the newly potentiated synapses above noise or ‘background’. In this scenario, a limited number of synapses would potentiate and carry new information, but an overall depression would be observed in extracellular field or whole-cell measurements that record populations of synapses. Such a form of synaptic depression would be functionally distinct from purely homoeostatic mechanisms, as it would contribute to information coding.

## 8. Future directions

To fully investigate the subunit selectivity hypothesis, priorities for the future should include the development of tools to selectively block GluN2A subunit-containing NMDARs. In addition, establishing techniques to target triheteromeric NMDARs is fundamental to investigate their role; it appears likely that some of the confusion in the current literature may result from the differing extents to which these receptors have been affected by the manipulations used. Combining subunit labelling with fluorescent probes and high-resolution live imaging could help to study this population in single spine-based plasticity studies. Alternatively, if a posttranslational modification, such as a disulfide bridge, could be introduced to link GluN2A and GluN2B subunits without disrupting their kinetic properties, this would help investigation at the level of whole-cell or extracellular field recordings. Given that a number of studies have suggested that the triheteromeric population may be substantial, or even dominant, in the adult brain, it is likely that this NMDAR composition has an important function. This may be because triheteromers have intermediate channel kinetics, and so combine the faster response and integrative properties provided by the GluN2A subunit with the important intracellular associations conveyed by the GluN2B subunit—in particular allowing Ca^2+^ influx to be closely coupled to downstream signalling molecules, such as CaMKII.

In order to understand whether the links between subunit involvement in plasticity paradigms and behaviour are meaningful, it is important to demonstrate conclusively that changes in synaptic weights can support learning and memory. This could then be dissected further to establish the types and synaptic location of plasticity that support behavioural tasks with different cognitive demands. In the past, technical limitations have prevented interrogation of a causal link between plasticity and learning and memory. However, new advances may help us test this hypothesis more directly. As the neural code is not yet resolved, driving learning to guide a complex behaviour by replacing experience with artificially introduced, specific changes in synaptic weights may not be on the immediate horizon, although significant steps in this direction have recently been taken [126]. Other approaches may still be fruitful: the newly developed optogenetic and pharmacogenetic technologies can be used to target synapses with distinct molecular and structural compositions and, when used in combination with molecular trickery, are likely to be a particularly powerful way to investigate whether different types of synapse play distinct computational roles. Such acute manipulations have already revealed new information about hippocampal function that differs from what had been suggested by pharmacological lesion studies [127]. Furthermore, given the technological advances in wireless recording devices, it will be important to record postsynaptic responses *in vivo* during behaviour to provide a read-out of the manipulations made. Such recordings would also show the extent of synaptic weakening in reversal tasks. In terms of the subunit-selectivity hypothesis, the current evidence suggests that the extent to which each subunit is involved in plasticity is affected by the induction protocol used owing to their distinct kinetics. Therefore, to understand the importance of GluN2A and GluN2B in physiological conditions and in relation to learning and memory, emphasis must be placed on investigating the natural activity patterns that occur during behaviour and which drive long-lasting changes in synaptic strength.

However, it should be remembered that NMDARs do not only play a role in plasticity, but also in slow or tonic response components of neural activity; for example, they have been shown to support slow responses in the thalamus and sensory tuning in the neocortex. Moreover, NMDARs may not even be required for certain forms of learning; it has recently been shown that mice with NMDAR knock-out in the DG and dorsal CA1 can still perform tasks typically considered to be NMDAR-dependent, for example the MWM [121]. This does not mean that, under normal circumstances, NMDARs are not involved in this type of learning, as other mechanisms or hippocampal regions could take over following prolonged deletion. Nevertheless, it will be important to investigate whether NMDARs at all hippocampal synapses are involved in learning and memory or, alternatively, whether only a subset is, while the rest of the hippocampal NMDARs perform computations similar to those in sensory neocortex. The hippocampal asymmetry reported in humans [128] and, more recently, in the mouse [69,94,96], makes it possible that the hippocampus consists of parallel networks, one being primarily a response network involved in sensory coding and navigation, whereas the other supports learning of associations. Despite the huge advances in our understanding of hippocampal function, it seems that there is still much more to uncover.
